# Parallel Driving with Big Models and Foundation Intelligence in Cyber–Physical–Social Spaces

**DOI:** 10.34133/research.0349

**Published:** 2024-06-06

**Authors:** Xiao Wang, Jun Huang, Yonglin Tian, Chen Sun, Lie Yang, Shanhe Lou, Chen Lv, Changyin Sun, Fei-Yue Wang

**Affiliations:** ^1^ School of Artificial Intelligence, Anhui University, Hefei, China.; ^2^ Macau University of Science and Technology, Macao, China.; ^3^ State Key Laboratory for Management and Control of Complex Systems, Institute of Automation, Chinese Academy of Sciences, Beijing, China.; ^4^ MVSLab, Department of Mechanical and Mechatronics Engineering, University of Waterloo, Waterloo, ON N2L3G1, Canada.; ^5^ School of Mechanical and Aerospace Engineering, Nanyang Technological University, Singapore, Singapore.

## Abstract

Recent years have witnessed numerous technical breakthroughs in connected and autonomous vehicles (CAVs). On the one hand, these breakthroughs have significantly advanced the development of intelligent transportation systems (ITSs); on the other hand, these new traffic participants introduce more complex and uncertain elements to ITSs from the social space. Digital twins (DTs) provide real-time, data-driven, precise modeling for constructing the digital mapping of physical-world ITSs. Meanwhile, the metaverse integrates emerging technologies such as virtual reality/mixed reality, artificial intelligence, and DTs to model and explore how to realize improved sustainability, increased efficiency, and enhanced safety. More recently, as a leading effort toward general artificial intelligence, the concept of foundation model was proposed and has achieved significant success, showing great potential to lay the cornerstone for diverse artificial intelligence applications across different domains. In this article, we explore the big models embodied foundation intelligence for parallel driving in cyber-physical-social spaces, which integrate metaverse and DTs to construct a parallel training space for CAVs, and present a comprehensive elucidation of the crucial characteristics and operational mechanisms. Beyond providing the infrastructure and foundation intelligence of big models for parallel driving, this article also discusses future trends and potential research directions, and the “6S” goals of parallel driving.

## Introduction

With the wide application of sensing technologies and pervasive use of location-based services, the social need-driven mobility dynamics not only can be documented but also can be analyzed and predicted for better traffic management and control. Thereupon, research on data-driven traffic congestion analysis, traffic lights control, traffic flow prediction, traffic situation sensing, etc., received growing attention in recent years and significantly improved the performance of intelligent transportation systems (ITSs). Not withstanding this progress, the launch of different levels of connected and autonomous vehicles (CAVs) further increased the diversity, heterogeneity, and complexity of urban traffic.

People have been seeking methods for improving vehicle intelligence for efficiency, safety, reliability, and environmental sustainability since the very first automobile was invented around 1885 [1]. However, every upgrade iteration in vehicle intelligence triggered some new problems while solving several old ones. One of the main causes is that we never have the chance to pretest how will the upgraded vehicles operate in and work with the old traffic systems [2–6].

Scholars began to explore the possibility of creating a digital-vehicle proving ground for field testing of CAVs since the early 2000s [7]. However, the progress in this direction has been very slow due to the limitation of computational power, modeling techniques, and digital–physical interfaces in the first 15 years of the 21st century. This situation has greatly changed in the few past years. Waymo used Carcraft and Castle to train its vehicles, in which Waymo says it has driven over 5 billion miles. Recently, Waymo published Simulation City, a virtual world where its autonomous vehicles are tested and trained in preparation for real-world experiences [8]. Intel launched its CARLA project in 2015, which is an open-source platform and went to public after the 2017 Conference on Robot Learning. CARLA supports flexible specification of sensor suites, environmental conditions, full control of all static and dynamic actors, maps generation, and so on [9]. NVIDIA DRIVE Constellation was introduced at the 2018 GPU Technology Conference to provide safe driving and testing solutions in virtual reality. It now covers everything from the car to the data center. Not only the function and intelligence of CAVs can be trained and tested, but also how the CAVs interact with other traffic participants and what changes will be brought to the traffic can be modeled and evaluated before they are launched.

Two of the vital concerns when training the vehicles in virtual environments are as follows: (a) How to ensure the vehicles perform in the physical environment as favorably as it was trained in the virtual environment and (b) How to enable the trained vehicles to deal with all kinds of safety-critical scenarios even with the ones that it has never been met before. For the first concern, high-precision traffic environment construction methods such DT-based modeling were proposed. DTs captured significant attention in recent years [10–13]. The decoupling and mapping approach prominently improved the modeling precision of traffic environment, but lacked consideration about the intention of and interaction among other traffic participants. Metaverse integrates artificial reality, virtual reality, and mixed reality to create immersive training space for retrofitting, redesigning, and even redefining the physical systems while providing humans with interfaces to interact directly with the virtual and immersive environment [14, 15]. Embedded with the lately bodacious artificial intelligence generated content (AIGC) technologies and AIGC tools [16–21], metaverse will be able to generate multi-kinds of safety-critical scenarios and provide much more abundant training scenes for CAVs [22–26].

Although the technical developments in DTs and metaverses greatly facilitated the construction and generation of training environments for CAVs, we cannot help but wonder if the CAVs will be able to interpret and understand the environment in which they are trained and are driving? And even proactively make social interactions with others when necessary? How should autonomous vehicles handle zero-shot problems? The emergence of large language models (LLMs) inspires such queries. LLMs employ human-in-the-loop reinforcement learning (HRL) [27] to update their models with interpretability [28–32], and thus endowed the capability to interact naturally with the physical world [17–19, 33]. Their integration with CAVs ensures interpretability, allowing for the direct generation of low-level control commands backed by reasoning.

In this paper, we explore how to integrate DTs, metaverses, and big models to construct the infrastructure and foundation intelligence of parallel driving, which aims to realize the “6S” (smart, safe, secure, sensitive, sustainable, and serviceable) goals of CAVs. The state-of-the-art research progresses in deploying DTs, metaverses, and big models for enhancing CAVs’ intelligence are discussed in the “Parallel Driving Architecture under Mixed Traffic and Its Challenges” section, the edge computing technologies supported in-vehicle big model is designed and presented in the “Big Models and Edge Computing inside Vehicles” section, while the federated computing and blockchain accredited vehicle-to-vehicle (V2V) foundation intelligence are clarified in the “Foundation Intelligence and FC Cross Internet of Vehicles” section. The “6S-Oriented Utility Evaluation of Parallel Driving” section illustrates the “6S” goals of parallel driving. In the end, we shared our perspectives on future prospects and research directions of CAVs.

## Methods

### Parallel driving architecture under mixed traffic and its challenges

The deep integration of human needs, driving styles, emotions, etc. and vehicle management and control with multimodel data fusion (MDF) promotes profound changes in driving patterns, which is generally called human–vehicle co-drive [34]. Benefiting from MDF and the internet of vehicles, the situations both inside and outside the vehicle can be sensed and awarded. However, due to the increasing number of traffic participants, the diversity of ways they participate in urban traffic, and the irregularity of travel behaviors triggered by different kinds of social activities, the traffic system becomes unprecedentedly complex and dynamic, which in turn increases the risk of accidents.

Parallel driving [35–39] is proposed for vehicles to handle the cyber–physical–social dimension integrated and fused traffic situations. It is the application of ACP-based parallel intelligence into intelligent vehicles. The ACP approach [40] is the extension of scientific research process of identifying a problem, proposing a hypothesis, reasoning and testing the hypothesis, and deploying the testified hypothetical results to the corresponding interested situations through a cyber–physical–social integrated comprehensive space. For our humans, once a problem is identified, we search the problem-related knowledge in our mind and collect the resources at hand, model how to solve the problem by designing a process to operate with the resources by thinking, which is generally called proposing a solution, and test the solution by executing the designed process. Now, with the emergence of LLMs, combined with embodied intelligence, mobile robots like CAVs are enabled the potential to drive themselves like a human.

**Fig. 1.**
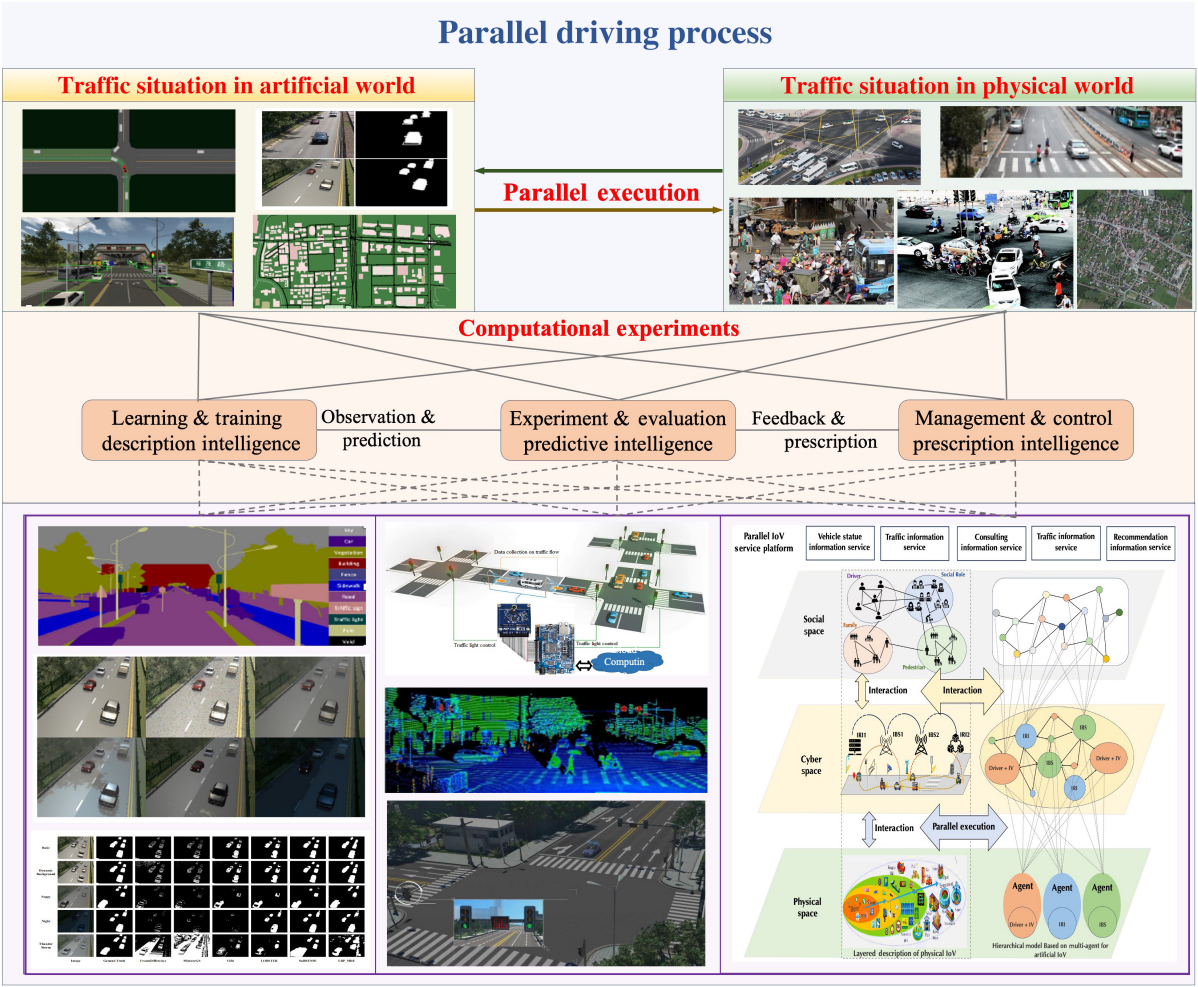
ACP-based parallel driving scheme.

In ACP, “A” represents artificial system (AS), which is the mapping of the actual physical system (APS), and is constructed by bottom-up multi-agent modeling methods, while knowledge and rules about how the APS operates are stored and distributed in each agent. “C” represents computational experiments. Each experimental environment demonstrates a hypothetical situation, which is designed for training and testing the performance of agents. Agents in ASs search the problem space in a distributed and autonomous organizational way, and propose solutions by trial and error via computational experiments. “P” represents parallel execution, which emphasizes the solution deployment in both APS and AS, to keep the parallel systems operating in synchronization. Thus, once the systems do not operate synchronously, a new problem is identified, and the process is reoriented to the ACP problem-solving circle. The ACP-based parallel driving scheme is illustrated in Fig. 1.

Earlier this year, the release of ChatGPT instantly drew the attention of artificial intelligence (AI) practitioners all over the world. The technical breakthroughs in LLMs further inspired intelligent vehicle practitioners to implant LLMs to human–vehicle interfaces [16–19, 41] for more natural and fluent cooperation of drivers and vehicles. In this part, we explore the integration of DTs, metaverses, and LLMs into parallel driving to realize the “6S” driving within ITS.

#### Mobility digital twins for description intelligence of parallel driving

Digital twins (DTs) is an advanced technology and concept that creates a digital replica or model of a physical entity or system and keeps it synchronized with the real object or process. Scholars [42] proposed the concept of mobility digital twin (MDT) to name the application of DTs in the modeling, simulation, and optimization of urban and transportation systems. MDT consists of three aspects: vehicle digital twin (VDT), driver digital twin (DDT), and traffic digital twin (TDT).

The VDT represents a data-driven emulation of a physical vehicle, comprising integrated physical sensors within the vehicle and corresponding digital data hosted in the cloud or edge network. It serves as the fundamental underpinning for comprehending, regulating, and optimizing the conduct and interplay of vehicles within a transportation system. Concurrently, cameras, radar, and LiDAR apparatuses perceive traffic conditions in conjunction with the situational awareness of proximate vehicles. These collective and multimodel data are transmitted to the digital space via the communication plane. Within this digital domain, the data undergo a series of distinct processes, including storage, modeling, learning, simulation, and predictive modeling, culminating in the formalized instantiation of the digital counterpart of the ego vehicle.

A DDT system consists of four parts: real driver, digital driver, multimodal interface, and related application. The real driver is the physical entity and basis of the DDT system. It is both the data generator and the recipient of system services. Whether in a semi-autonomous driving system or a fully autonomous driving system, humans will always play an important role and coexist with vehicles [43, 44]. Digital driver is a virtual replica of the human driver. It can realistically, comprehensively, and synchronously reflect the behavior of the real driver. During a travel, the digital driver model will interact with the human driver in real time to monitor the emotional fluctuations and ability degradation of the real driver.

In MDT, the TDT serves as a dynamic representation of road infrastructures and traffic conditions within a specified geographic area. It is crafted through the assimilation of real-time data generated or measured by various traffic infrastructures, encompassing traffic lights, roadside units, and detectors [45]. This amalgamation results in a high-fidelity DT that mirrors the physical layout of the traffic system.

**Fig. 2.**
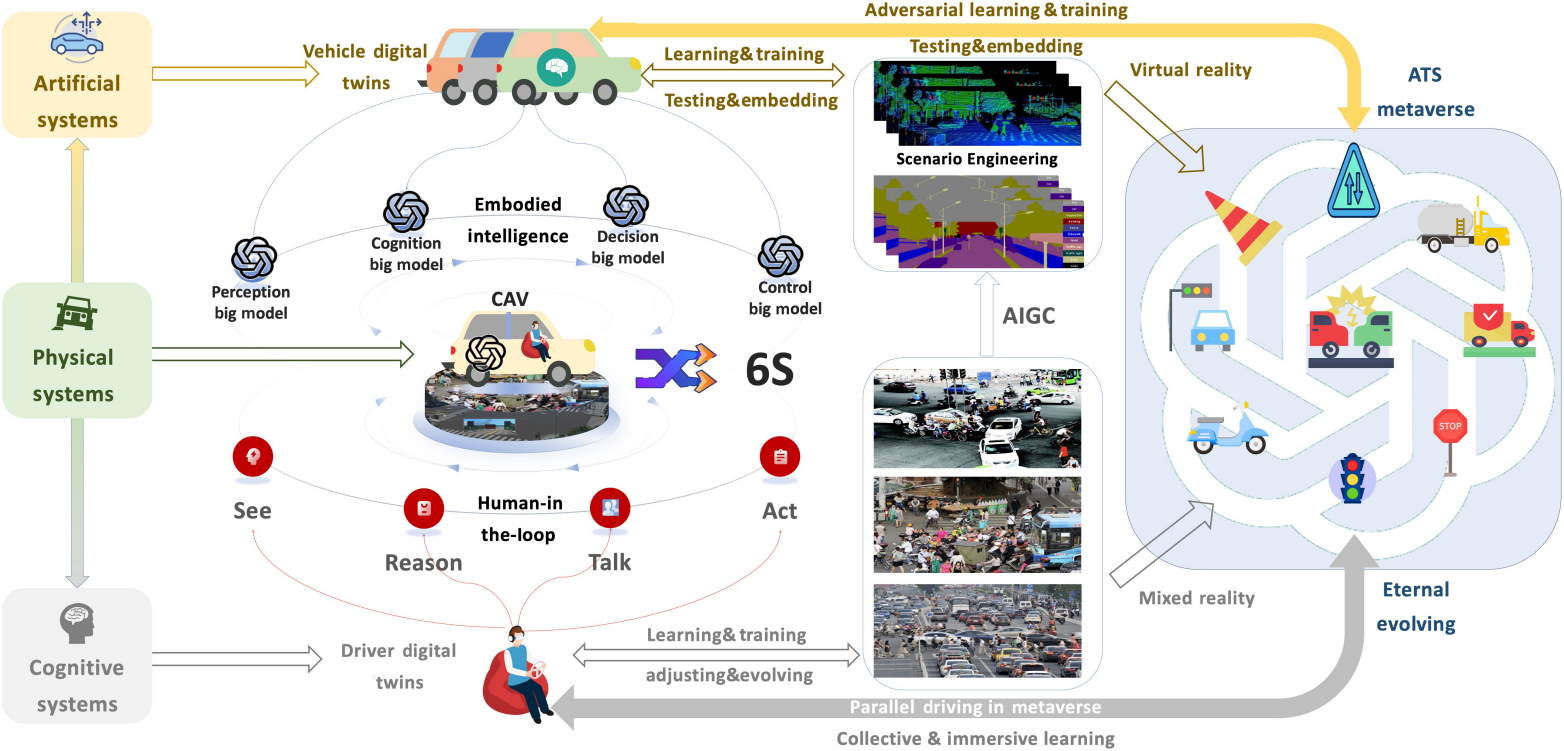
Parallel driving with big models and foundation intelligence.

MDT describes vehicle mobility in ITS at the individual level, while artificial transportation systems (ATSs) consider traffic management and control in system level. Each MDT can be seen as a module in ATS [46], which lays the foundation to model the whole ITS. However, there are other participants such as pedestrians, cyclists, and motorcyclists in mixed traffic. Each kind of participant can be represented by one type of agent with specific properties, following specific rules and exhibiting joint causality between the individual level and system level. In this manner, we can not only model the participants in self-centered and serving ways but also describe the altruistic manner of human behavior (Fig. 2).

#### Metamobility for prediction intelligence of parallel driving

Metamobility presents an emerging conceptual framework that embodies the holistic integration of the metaverse (“meta”) within the realm of mobility [45]. The primary objective of metamobility is to manifest a seamless, enjoyable, and personalized mobility ecosystem, underpinned by two pivotal attributes. (a) Overcoming spatial and temporal constraints: Metamobility seeks to transcend the inherent limitations of physical space and time, allowing for a fluid and adaptive mobility experience. (b) Cyberspace-reality synchronization: It endeavors to ensure that modifications made within cyberspace are accurately and promptly reflected within the tangible mobility system.

To realize metamobility, the data harvested from the physical world adhere to a crowdsourcing paradigm [47], capitalizing on the ubiquity of smart sensors integrated into both stationary and mobile entities. Edge-cloud computing technologies [48] are instrumental in alleviating the computational burdens associated with processing vast datasets while enhancing the performance of latency-sensitive and computation-intensive mobility applications. Furthermore, the integration of trustworthy AI, DT, and blockchain technologies within cyberspace serves to fortify the foundation of reliable and robust data-driven analysis, simultaneously safeguarding against potential privacy breaches.

Since AIGCs become a new generation engine of digital content, the integration of AIGC tools into the metaverse will significantly enrich the diversity of traffic scenarios in the digital world. On the one hand, virtual contents of rare cases from the physical world can be generated, used for training and testing the performance of CAVs [49] in safety-critical scenarios; on the other hand, even the traffic situations that never happened before can be generated, which enables CAVs to imagine what can happen in the future, thus get pretrained and prepared.

Extended reality (XR) techniques further facilitate the seamless interaction and immersion of human users within the cyberworld, permitting the acquisition of immersive experiences derived from real-world scenarios. Tactile live maps exemplify a captivating metamobility application that enhances the perception of the physical world, facilitating immersive engagement with virtual content [45]. Leveraging the aforementioned technologies, this transformative concept enables real-time traffic information updating, immersive navigation experiences, personalized intelligent cockpit, and engaging in-vehicle gaming.

#### Embodied big models for prescription intelligence of parallel driving

Embodiment is a crucial concept for the autonomy and adaptivity of systems working in the physical world with high degrees of uncertainty and complexity [50]. Embodied intelligence is the computational approach to the design and understanding of intelligent behavior in embodied and situated agents through the consideration of the strict coupling between the agent and its environment, mediated by the constraints of the agent’s own body, perceptual and motor system [51], and brain (embodiment) [52]. Big models, such as LLMs, and foundation graph models, have been shown to possess a wealth of actionable knowledge that can be implanted into physical devices for manipulation in the form of reasoning and planning [53]. Embodied big models for prescription intelligence of parallel driving aim to train and enable agents’ ability to see (vision), talk (language), act (behavior) such as navigate and interact with their environment, and Reason (Thought), by combining and integrating different kinds of large models to a CAV.

In an urban traffic system, each CAV can be seen as an agent with embodied intelligence, which works and drives in the dynamic and complex mixed traffic. These agents can vary in types, shapes, and functionalities. Taking CAV as an example, it “sees” its situation through data collected from devices such as cameras, radar, and LiDAR and transmits them to the cloud and other CAVs in the same ecosystem. However, a standalone CAV typically lacks the capability to discern the social behaviors associated with driving on public roads, thereby impeding the realization of high-level autonomous driving in public road scenarios. In mixed-traffic situations, understanding the behavior or intentions of other vehicles and interacting with them safely and efficiently is a huge challenge for the field of autonomous driving [35]. Wang [54] thus proposed the parallel driving architecture to accomplish the “6S” driving goals of CAVs.

Under the artificial–physical interactive and co-evolutional architecture of ACP, parallel driving employed DTs for ATS construction, while integrating the embodied big models with multi-agent modeling, enabling each agent in ATS the ability to see, talk, act, and reason, and embedded AIGC tools with the metaverse to generate diverse and abundant scenarios by scenario engineering through computational experiments. Agents and the embodied models are trained and tested using different kinds of generated data via computational experiments, and once a promising big model has been trained and tested, it can then be deployed into the actual vehicles.

In recent years, numerous researchers have explored and developed critical technologies in parallel driving, and designed plenty of subsystem structures under it. Liu et al. [55] proposed a parallel driving system based on DTs, which consists of description vehicles, prediction vehicles, guidance vehicles, and real vehicles. This system provides sufficient testing and optimized decision-making schemes for autonomous vehicles through artificial scenes, large-scale computational experiments, and online learning and guidance to ensure the safe and stable operation of autonomous vehicles. To guide the accurate perception of road environment information, Zhang et al. [24] proposed a parallel vision framework (PVITS), which consists of virtual traffic space construction, model learning based on computational experiments, and feedback optimization based on parallel execution. Inspired by the parallel vision theory, Chen et al. [56–58] proposed an end-to-end parallel planning framework, which makes autonomous driving vehicles capable of driving like humans and effectively handling emergencies. Li and his colleagues [30] designed a parallel testing method, which implements more challenging tests to accelerate the inner ability construction of CAVs via autonomous generation of tasks. These studies are conducive to the implementation of parallel driving theory in the application scenarios, which are of great significance in addressing the complexity and challenges in the field of autonomous driving.

## Results

### Big models and edge computing inside vehicles

**Fig. 3.**
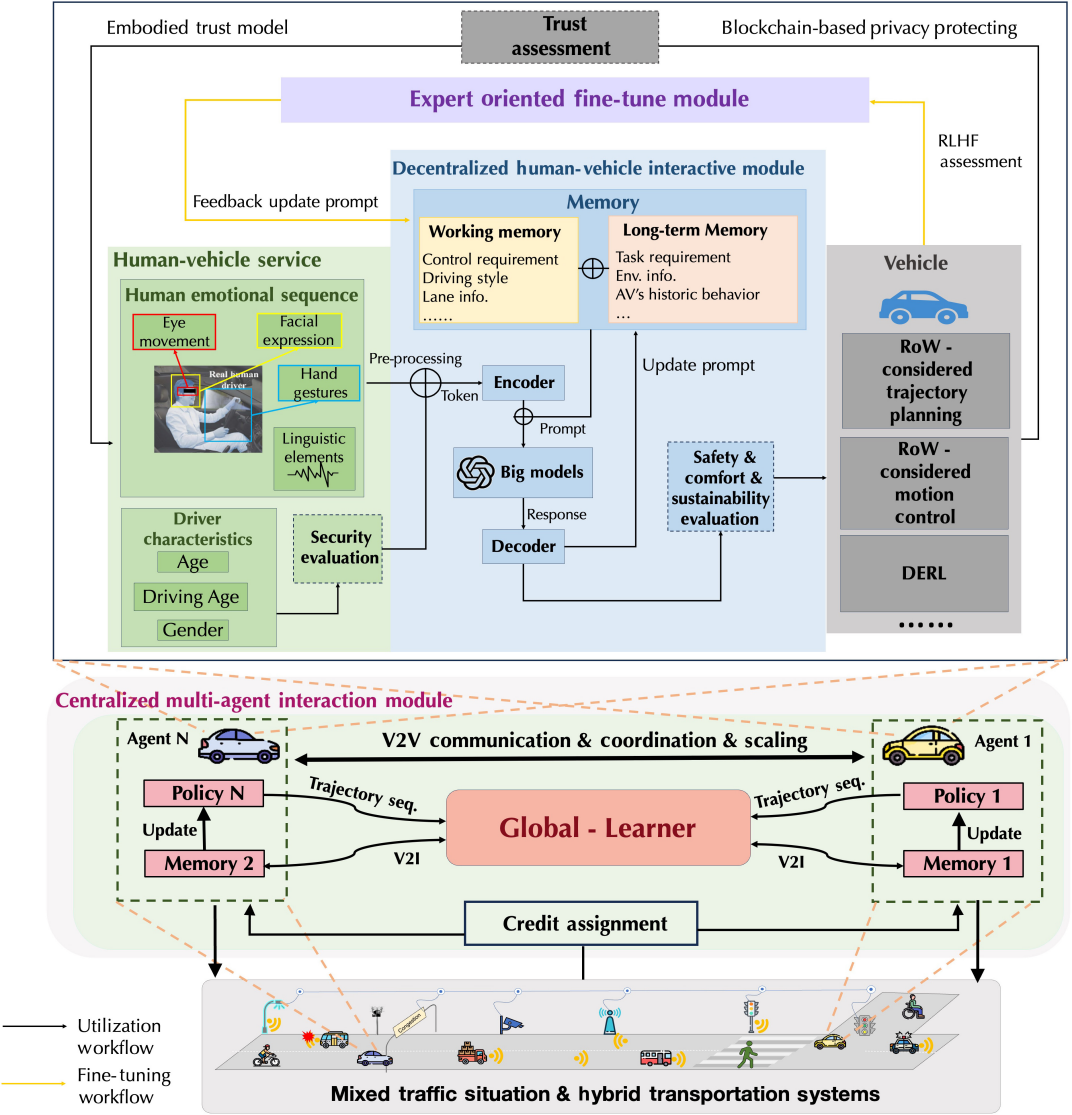
The human–vehicle interactive framework.

ATS is a pivotal element in parallel driving systems, aiming to revolutionize traffic management and control on a systemic level [37, 40, 59]. The essence of ATS is to provide a comprehensive framework that encompasses vehicular dynamics and the interactions among all road users, including pedestrians, cyclists, and motorcyclists, thus ensuring a multifaceted approach to understand and optimize mixed-traffic situations for increased efficiency and safety. Central to ensuring traffic efficiency and safety within this comprehensive framework is the identification of the driver’s state in the physical world. Recognizing this critical element, the focus of this section is on developing a human-vehicle interaction framework that delves into an in-depth analysis of the driver’s abilities, present condition, and behavioral tendencies. This framework aims to construct a real-world driver’s characteristic and reaction replication through the DDT, a sophisticated digital model that mirrors these crucial aspects of the driver. By integrating ACP technology, this approach becomes instrumental in processing extensive datasets and broadening the system’s application range. The synergy of big models with edge computing within this framework is pivotal for generating decision-making processes that are not only aligned with the driver’s driving style but also profoundly informed by their broader behavioral and intentional context [33]. Through an expert-driven fine-tuning process, the model’s final decisions and controls are meticulously calibrated to align closely with human intentions, as illustrated in Fig. 3. Furthermore, this section will elaborate on the two primary workflows of the model, detailing the functions of the principal modules. Through this comprehensive exploration, the integration of the DDT within the ATS framework underscores the critical role of accurately identifying and replicating the driver’s state for enhancing the interaction between the vehicle and the driver. This endeavor not only aims to reflect actual driving scenarios more accurately but also strives to advance the overarching goals of ATS in creating a more adaptive, intelligent, and safe transportation system that effectively accommodates the complex dynamics of mixed-traffic environments.

#### Human-vehicle interactive channels and model

The crux of enhancing human–vehicle interaction lies in achieving seamless communication between humans and CAVs. The interface between DDT and VDT forms the crux of this engagement. The entire framework consists of four main components: human–vehicle service, human–vehicle interactive module, the vehicle, and the expert-oriented fine-tune module.

Human–vehicle service: This service is pivotal for considering the emotional state of the driver, to synergize their preferred driving style with the vehicle’s operational protocol, resulting in an adaptively planned trajectory and thoughtful control strategy. To achieve this understanding, we employ an advanced multimodal data acquisition system that captures various behavioral cues. Eye movement tracking, hand gesture recognition, and facial expression analysis are integrated with linguistic analysis to construct a comprehensive picture of the driver’s current emotional landscape. Each modality is meticulously processed using tailored deep-learning networks designed to function as sophisticated tokenizers. These networks transcribe real-time sensory data into a universal language of tokens, which are then fed into the encoder for further nuanced interpretation. Furthermore, we recognize the importance of personal background variables including age, social role, and gender in adjusting emotional response and driving behavior [60–64]. Our model makes allowances for these factors to ensure that our emotional regulation framework is not only reactive but also predictive and personalized.

Decentralized human–vehicle interaction module: This module’s sophisticated interface harnesses multimodal data to understand and adapt to the emotional and cognitive states of drivers, thus ensuring a safer and more personalized driving experience.

Encoder: Upon receiving tokenized signals from the human–vehicle service and memories, the encoder contextualizes these inputs, effectively turning raw multimodal data into meaningful prompts. It employs a series of layers that encapsulate convolutional and recurrent neural transformations, which ultimately distill complexity into actionable insights for the big models.

Memory: The concept of memory plays a pivotal role in the cognitive architecture of language agents [65]. This architecture enables the organization of information into multiple memory modules, each tailored to capture a different spectrum of knowledge. Drawing from psychological theories, the architecture delineates memory into two primary categories: working memory and long-term memory, which are essential for decision-making processes within CAVs.

Working memory holds information on current control requirements, the driver’s distinctive driving style, and pertinent lane information. This memory also contains the agent’s goals and the outcomes of internal cognitive processes that encapsulate intermediate reasoning. The calculation process is as follows:

$$f_{\mathrm{WM}}=F_{\mathrm{WM}}\left(\;f_{\mathrm{cc}},f_{\mathrm{ds}},f_{\mathrm{li}},f_{ae},f_{\mathrm{cog}}\right)$$ (1)

where *f_cc_*, *f_ds_*, *f_li_*, *f_ae_*, and *f_cog_* represent the current control requirements, distinctive driving style, pertinent lane information, agent’s expectations, and outcomes of internal cognitive processes, respectively.

Long-term memory serves as a unified repository that encapsulates a comprehensive set of behavioral directives, environmental knowledge, and historical data, thereby forming the cornerstone of vehicular intelligence. Within this module, procedural rules derived from the working memory guide adaptive responses to real-time vehicular status and situational demands, aligning actions with driving style preferences and road positioning. In conjunction, it integrates semantic knowledge, including traffic regulations and patterns, alongside context-specific insights, allowing for informed strategy formulation. Additionally, by combining past navigational experiences, the memory system yields a reflexive dimension, where historical behaviors inform current decision-making and facilitate continuous refinement of vehicular operations. This intricate melding of past and present data streams within the long-term memory furnishes the autonomous agent with a sophisticated cognitive framework essential for navigating complex driving environments. The calculation process is as follows:

$$f_{\mathrm{LTM}}=F_{\mathrm{LTM}}\left(f_{\mathrm{bd}},f_{\mathrm{ek}},f_{\mathrm{hd}},f_{\mathrm{tr}},f_{\text{rule}}\right)$$ (2)

where *f_bd_*, *f_ek_*, *f_hd_*, *f_tr_*, and *f_rule_* represent the behavioral directives, environmental knowledge, historical data, traffic regulations and patterns, and procedural rules derived from working memory for adaptive responses, respectively.

Thus, the formulation for the complete memory module can be expressed as:

$$f_{\mathrm{MM}}=\text{concat}\left(f_{\mathrm{WM}},f_{\mathrm{LTM}}\right)$$ (3)

Big models: Prompts derived from the memory module and encoder are conveyed to embedded big models via application programming interfaces (APIs). It is noteworthy to mention that the big models utilized in our research are the standard, out-of-the-box variants equipped with their original configurations; no additional tuning or task-specific adjustments are made prior to their implementation, ensuring that the models’ responses are solely the result of their inherent capabilities and general training.

Decoder: Upon receipt of a response from the LLMs, the decoder interprets this information by a predefined response pattern. Typically, this entails translating the high-level response, often a detailed command, into actionable instructions suitable for the vehicle’s operational context. These instructions are then relayed to the vehicle’s low-level control module, achieving the desired outcome in the vehicle’s behavior.

Safety, comfort and sustainability evaluation: Considering that the big models are inherently probabilistic, their application of CAV’s control and trajectory planning tasks presents potential safety hazards. To mitigate the risks posed by illusionary phenomena inherent in big models, a dedicated safety and comfort evaluation module is essential within the operational workflow. This module serves as a critical checkpoint, meticulously evaluating the safety and comfort levels of the outputs generated by big models before their implementation.

Strategically embedded in the workflow, the safety and comfort evaluation module functions at decisive stages, either after processing the responses from big models or just before the CAV’s systems execute control commands. Its fundamental aim is to rigorously analyze proposed actions, ensuring that they do not stray from established safety thresholds or comfort standards. This thorough scrutiny of potential risks and the alignment of big model-generated outputs with recognized driving safety norms are vital. It guarantees that all operational decisions prioritize driver and passenger welfare, thus fortifying the safe deployment of AI-informed directives in vehicular navigation and control systems. The sustainability evaluation module is a critical addition to the CAV framework, designed to complement the safety and comfort evaluation by assessing the environmental impacts of vehicular decisions. It measures and optimizes for factors such as emissions, energy consumption, and route eco-friendliness, promoting operations that are not only safe and comfortable but also eco-efficient. By integrating this module, the system ensures that autonomous vehicles contribute positively to environmental sustainability, reinforcing the commitment to a greener future in transportation. Trust assessment: The CAV’s decisions and proposed actions are scrutinized to ensure that they align with the human operator’s expectations and safety standards. Once the trust criterion is satisfactorily met, the CAV then reiterates through the tuning process. By iterating through this cycle, the vehicle continually enhances its decision-making capabilities, ensuring that its control and trajectory planning are not only efficient and effective but also trustworthy and aligned with the human operator’s expectations and comfort. Notably, to achieve a system that is more responsive and aligned with human needs and values, we have applied the concept of decentralized autonomous organization (DAO) [66–69]. Unlike traditional end-to-end solutions, DAOs can handle complex tasks and provide a more flexible framework for integrating human feedback into the training model. This approach allows for a dynamic and adaptable interaction between humans and AI, fostering a collaborative environment where both can contribute effectively to the development and refinement of CAV systems.

Centralized multi-agent interaction module: In the context of modeling multi-agent interactions within mixed-traffic situations and hybrid transportation systems, the centralized multi-agent interaction module, as visualized in the accompanying figure, plays an important role. This module integrates a global learner, analogous to the actor-critic model [70–72], which takes the trajectory sequences generated by the vehicles as input and outputs updates to the contents of each CAV’s memory, subsequently refining the vehicle’s policy. This setup addresses the complexities of V2V communication, coordination, scaling, and credit assignment. The function of this module is to ensure that CAVs can adapt not only to scenarios that require explicit communication channels and cooperative interactions but also to competitive settings and scenarios where only physical interactions between agents occur. By situating the global learner centrally, it can process the aggregate information from multiple CAVs, enabling a more coordinated and globally aware approach to strategy development and trajectory planning. This centralization facilitates the emergence of globally optimized strategies, ensuring that CAVs can operate effectively in the dynamic and sometimes unpredictable environments characteristic of real-world traffic systems.

#### Expert-oriented fine-tuning

**Fig. 4.**
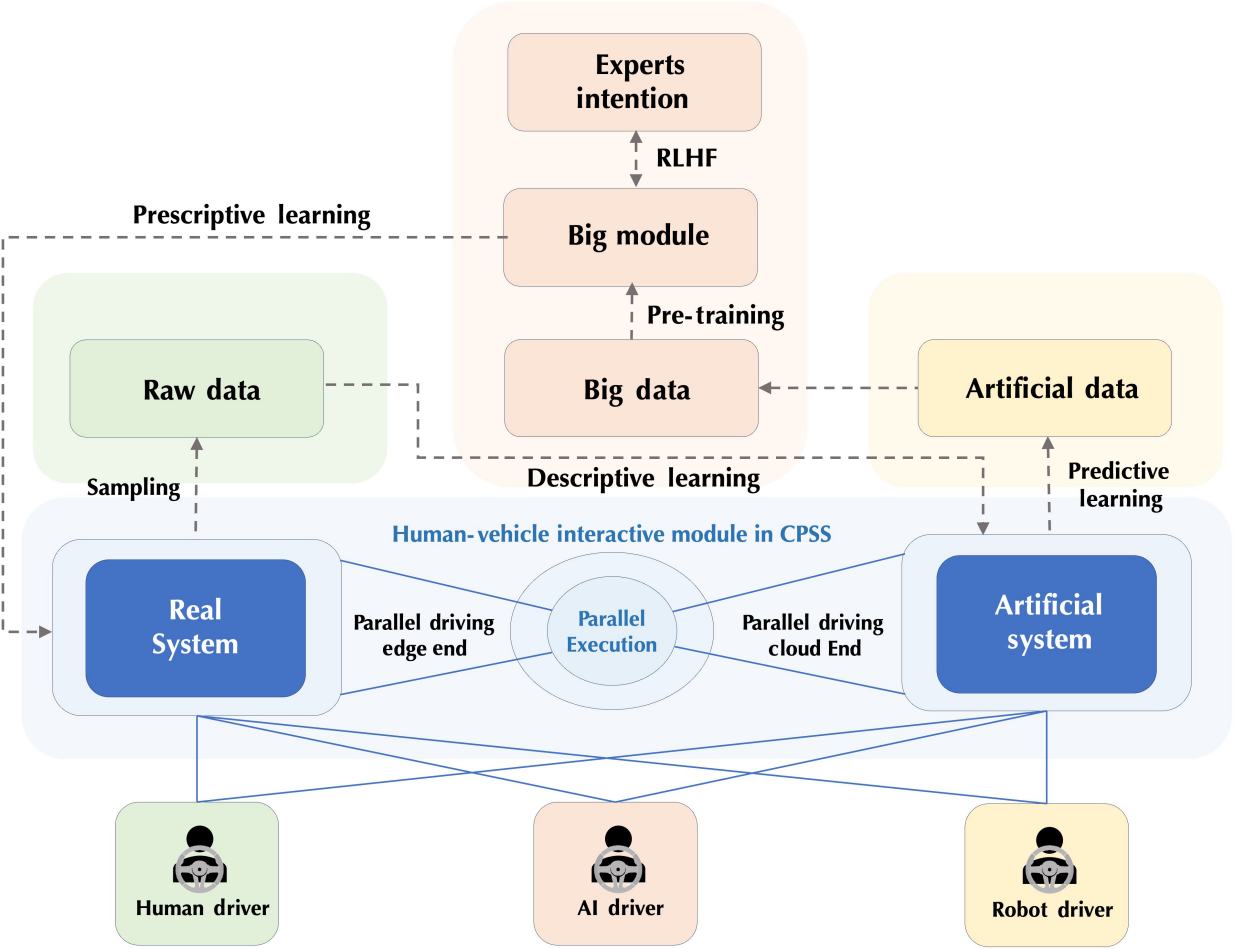
The parallel alignment framework of biological, digital, and mechanical drivers.

Under the architecture of parallel driving [3, 73], there are three distinct drivers operating within different spaces: the biological (human) driver in the physical-social domain, the digital driver in the social-cyber domain, and the mechanical driver in the cyber-physical domain, as depicted in Fig. 4. A digital driver is designated for each human operator, fostering a partnership by sharing sensory data and engaging in simulated “computational experiments” and “parallel executions.” This symbiosis enables the refinement of the digital driver’s capabilities until its intelligence is mature enough for its algorithms to be integrated into the mechanical driver. The autonomy of vehicle operation by the mechanical driver is contingent on the human driver’s discretion, offering a hybrid model of autonomous driving. This model maintains human engagement in oversight and decision-making processes, ensuring a collaborative approach to autonomous vehicular navigation. Building upon our integration of ACP parallel intelligence technology and the comprehensive framework of human–vehicle interaction channels and models, we introduce an innovative approach: human-in-the-loop feedback reinforcement learning (RLHF) [74–79]. This mechanism, rooted in the principles of RLHF, evolves into a sophisticated iterative workflow. It actively involves domain experts in the ongoing refinement and improvement of control strategies and trajectory planning for autonomous vehicle systems. This specialized fine-tuning process is structured into three distinct stages, each utilizing human expertise to methodically enhance the system’s overall performance and decision-making capabilities.

Assessment phase with RLHF: Domain experts initiate the RLHF process by thoroughly assessing the autonomous system’s capacity to perform control tasks and plan trajectories. This assessment is not only observational but also interactive, as experts provide direct, qualitative feedback on performance metrics, identifying shortcomings or deviations from intended outcomes. This forms the basis for the RLHF, where human judgment is used to reinforce desired behaviors and correct erroneous ones, setting clear objectives for the learning algorithm to achieve.

Update phase with expert feedback integration: With the insights gained during the assessment, the long-term memory, which is central to the vehicle’s decision-making framework, undergoes strategic updates. These updates, aligned with the feedback from the RLHF assessment, involve the integration of intricate explanations of key concepts, the introduction of context-rich examples into episodic memory, and the embedding of updated procedural knowledge. This phase endeavors to fortify the vehicle’s knowledge base, addressing the challenges inherent to zero-shot learning and ensuring an advanced understanding aligned with human reasoning.

Optimization phase with derivative-free learning: The expert feedback integrated during the update phase informs the subsequent derivative-free optimization within a specified subspace. The RLHF methodology comes into full effect now, leveraging reinforcement learning to fine-tune the system’s behaviors through a series of model iterations. The autonomous system’s performance is continually assessed and optimized against the fine-tuned long-term memory, allowing for adaptive improvement that is calibrated to expert feedback and real-world conditions.

By combining RLHF into the fine-tuning process, domain experts directly mold the learning trajectory of the big models. Such an approach results in a powerful blend of human intuition and machine learning, producing an autonomous system that not only mimics expert proficiency but also embodies a depth of understanding necessary for tackling complex driving environments and meeting rigorous safety standards.

In addition to the derivative-free optimization informed by expert feedback, the integration of deep evolutionary reinforcement learning (DERL) plays a pivotal role in enhancing the adaptability of human–vehicle interactive systems. DERL is a computational framework designed to evolve diverse agent morphologies for complex tasks in varying environments. It combines the principles of Darwinian evolution with reinforcement learning, enabling agents to learn intelligent behavior from low-level egocentric sensory information. A key aspect of the DERL framework is its use of distributed asynchronous evolutionary search, which parallels the computations underlying learning and leverages the scalability of computation and models. This approach effectively combines the evolutionary development of agent morphologies with reinforcement learning, equipping systems to adeptly handle complex driving tasks in varied environments.

Such capability is crucial for the rapid learning and adaptation of morphologies, underscored by the morphological Baldwin effect. This evolutionary dynamic ensures that autonomous systems not only mimic expert proficiency but also are inherently designed to evolve and adapt to new challenges in real time. As a result, the application of DERL could significantly enhance the capability of human–vehicle interactive systems to navigate and respond to the complexities in mixed-traffic situations and hybrid transportation systems. This aligns with the overarching objectives of safety, security, and sustainability in modern traffic management, ensuring that these systems not only are efficient and responsive but also adhere to the highest standards of safety and environmental consideration.

#### Prompt learning in SReTA

In the context of autonomous driving systems, the concept of embodied big models for prescription intelligence and the implementation of the SReTA (see, reason, talk, act) framework are significant advancements. They represent a significant shift from traditional OODA (observe, orient, decide, act) methodologies [80], allowing for more nuanced and responsive interaction between CAVs and their environments. Prompt learning enables pretrained models to adapt to new applications without additional training [75, 81–84] and adds a dimension of flexibility. By embedding natural language text cues into the pretraining phase, the model is prepared to recognize and respond to a wide range of scenarios related to human–vehicle interactions. In addition, building on the foundations of visual detection methodologies [85], the system not only captures the human–vehicle interactions we mentioned in the “Human-vehicle interactive channels and model” section, but also interprets the behavior of traffic participants such as human-driven vehicles (HDVs) and pedestrians in both real and virtual environments. This comprehensive view extends to discerning intricate traffic signals. The normalized detection bounding box coordinates, which refer to privileged information, are used to prompt the big models.

The SReTA framework is composed of four integral components, each contributing to a comprehensive and adaptive driving system. First, the “see” component utilizes advanced visual detection methodologies, enabling the system to assimilate and interpret environmental data, including the behaviors of various traffic entities like HDVs and pedestrians. This phase forms the foundation for situational awareness. Subsequently, the “reason” aspect employs sophisticated analytical tools and privileged information, such as normalized detection bounding box coordinates [86]. This step is vital in shaping the perception and cognitive understanding within the big models, ensuring that the data gathered in the “See” phase are effectively processed and contextualized. In the “talk” phase, the system harnesses the power of natural language processing, utilizing prompts to enrich the decision-making process. These prompts are pivotal in guaranteeing that the decisions executed by CAVs are both algorithmically robust and contextually attuned to the subtleties and complexities of human behavior. Finally, the “act” component translates the synthesized decisions into tangible actions. This involves executing precise vehicular maneuvers and adaptive controls, emphasizing safety, efficiency, and a seamless interface with human drivers. Through the integration of prompt learning within the SReTA framework, CAVs are equipped to interpret, react, and proactively adapt to the dynamic and complex scenarios of real-world driving environments. This approach not only enhances the responsiveness and safety of CAVs but also fosters a more intuitive and harmonious interaction between machines and humans, paving the way for the future of autonomous driving technology.

#### Human–vehicle collaboratively parallel driving in cyber–physical–social systems for right-of-way

Right-of-Way (RoW) is the primary factor for CAVs driving safely and smoothly in mixed traffic. As delineated in the paper, the integration of RoW within the TDT is a vital development, crucial for the dynamic and efficient decision-making processes required in mixed-traffic scenarios, where CAVs and HDVs coexist [87, 88].

Integrating the RoW with the TDTs ensures that decision-making is more dynamic, efficient, and adaptive to changing traffic situations. This is especially critical given that under general conditions, vehicles operate on the “first arrive, first serve” (FAFS) principle. Such an approach is computationally straightforward, but TDTs bring an additional layer of data that can provide context, allowing vehicles to make better-informed decisions. When vehicles adhere to this FAFS principle, they interact not in isolation but as part of a larger traffic ecosystem. It offers real-time insights into the road conditions, traffic flow, and movements of other participants, effectively integrating the DDT, VDT, and their interfaces as a coherent whole. This approach to RoW is not only rule-based but also dynamic, allowing the CAV to navigate complex driving scenarios safely and efficiently.

During the “See” and “Reason” phases, not only does the CAV capture environmental details like traffic signals, but through the TDT, it is also updated on roadworks, sudden congestion, or even emergency vehicle movement. This real-time data infusion ensures a broader and more updated understanding of the road environment. When the system proceeds to discern the road priority of each participant, it leans on this rich dataset, adding a layer of responsiveness and adaptability to its decisions. In the “talk” phase, the CAV, then equipped with human feedback, historical data, and real-time TDT insights, crafts its course of action. Whether it is yielding to a pedestrian who has decided to jaywalk or predicting the lane-changing intention of an HDV, the CAV’s decision-making is informed, timely, and dynamic. The subsequent “act” phase is enriched by the TDT’s input. The model, apart from traditional controls like speed regulation, may also adjust its trajectory based on real-time traffic flow or reroute altogether if an upcoming congestion is detected.

In essence, the integration of RoW with TDTs shifts the paradigm from a purely logical and algorithmic approach. It paves the way for a holistic, “human-centric” methodology where decision-making is informed by real-time traffic dynamics, ensuring a smoother interplay between human–vehicle and vehicle–vehicle integrations. This positions CAVs not as isolated entities but as adaptive participants in a complex traffic ecosystem. Simultaneously, by incorporating the principles of RoW into this intelligent framework, CAVs can make more nuanced decisions that respect the precedence of other road users while maintaining smooth traffic flow. This ability to interpret and react to the intentions of human drivers and other traffic participants in real time is essential for the integration of CAVs into the broader transportation ecosystem, ensuring that they contribute positively to the overall functionality and safety of the road network.

## Foundation intelligence and federated computing cross internet of vehicles

**Fig. 5.**
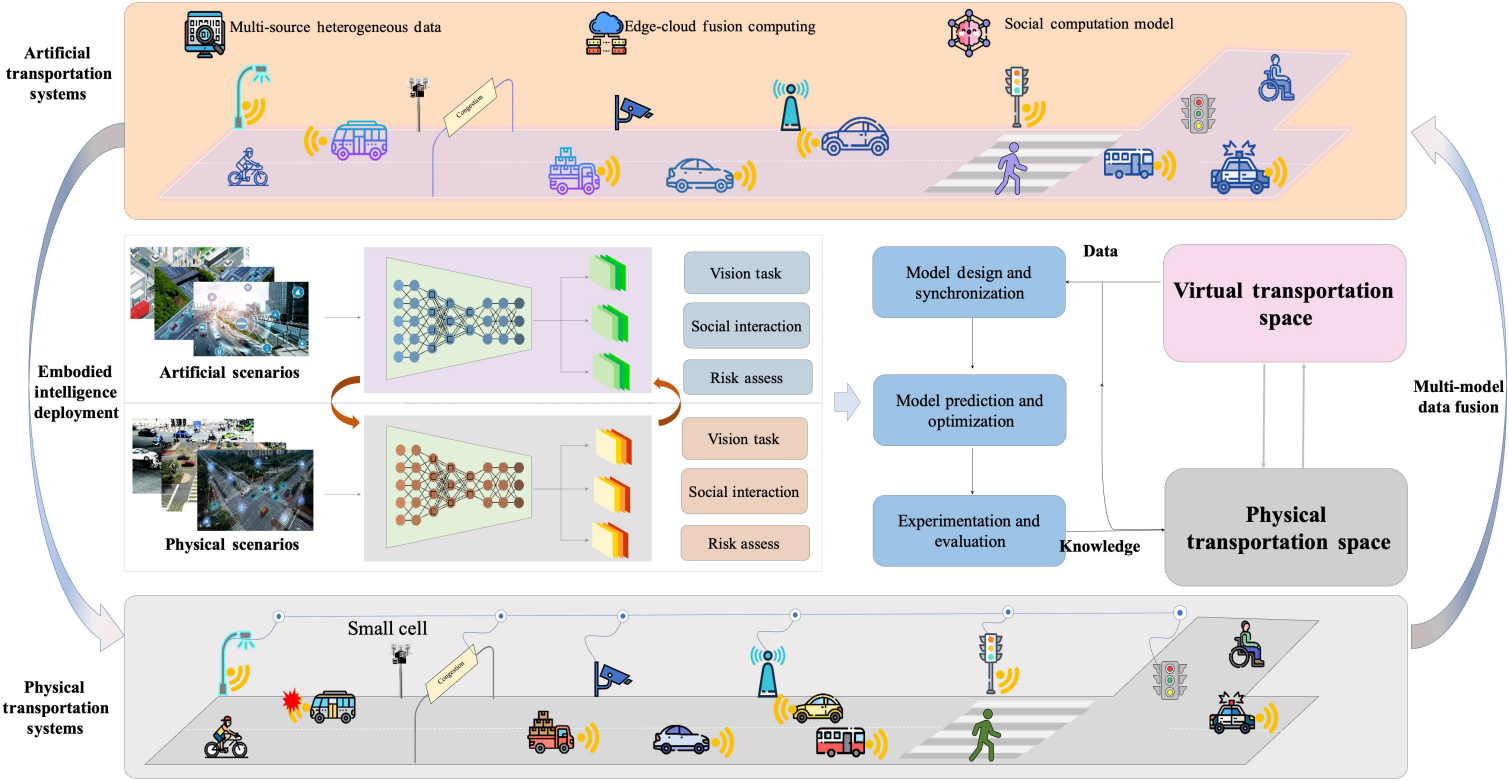
Foundation intelligence and federated computing cross internet of vehicles.

Urban transportation systems exhibit characteristics such as dispersed and hybrid elements (especially human-driven cars and autonomous cars), intricate and multidimensional relationships, and a high degree of coupling in social functions, which significantly increase the difficulty and complexity of urban traffic management and control. Foundation models have demonstrated distinguished generalization and transfer capabilities on the understanding of human emotion and interactions, thus holding significant potential to enhance the safety and efficiency of hybrid transportation systems [83]. Nevertheless, constructing such foundation models necessitates vast and diverse training examples as well as continuous validation mechanisms. Internet of vehicle (IoV) infrastructures provide an underlying platform for downstream applications or scenarios with the multimodal data from the individual cars and the communication supports between different cars, which greatly facilitates information sharing. To address the challenges in hybrid transportation systems and benefit the understanding ability of ITS for social factors, this section introduces a federated computing (FC)-based parallel IoV framework to effectively harness disparate data resources and build social-aware foundation models for the perception and prediction of hybrid transportation systems [89, 90]. We consider the task from three perspectives, i.e., data connection, resource utilization, and task orientation. First, we adopt parallel IoV for data connection, which bridges the gap between distributed sensors and utilizes a real-virtual interaction mechanism to generate large-scale training samples and lay the foundation for the improvement of social efficiency. Second, we adopt FC [91] for resource utilization, which achieves privacy-preserving data usage and is beneficial for social security. Third, we utilize foundation models for task orientation, which finetunes general models for specific tasks to transfer the common knowledge to a dedicated area and achieve social awareness. The framework, illustrated in Fig. 5, constructs a collaborative ecosystem where each participant contributes to the evolution of a socially aware foundational model based on parallel IoV systems, ensuring that the generated AI system can operate with a high degree of social intelligence within multifaceted traffic environments. The overall framework is as shown in Fig. 5.

First, to extend the scale and diversity of the IoV system, we adopt the parallel IoV [90] to incorporate both real and virtual AI agents [92] in hybrid transportation systems. Real agents refer to actual vehicles on the road, while virtual agents can be simulations, AI-driven vehicles, or even infrastructure elements like traffic lights and road sensors. By integrating these agents, we can gather data from a wide range of sources. Real agents, such as cars on the road, provide real-time data about traffic conditions, weather, and road hazards. Virtual agents can simulate various scenarios, helping us anticipate and respond to potential issues. For example, a virtual agent can simulate heavy traffic congestion and propose alternative routes to real agents, reducing congestion and improving overall traffic flow. To generate virtual vehicles, both data-driven methods, e.g., deep reinforcement learning, and physical-driven methods, e.g., intelligent diver model, can be used to clone and then extend the behavior patterns of real vehicles. With the parallel IoV systems, we can get access to the states and surrounding sensory data of real vehicles in a wide range and simultaneously reconstruct the environments and traffic conditions in virtual space to further simulate potential issues. These factors facilitate the design, optimization, and evaluation of foundation models by providing low-cost and flexible computing experiment platform.

To utilize the distributed data resources in a secure manner, we design the FC framework for IoV systems on blockchains. Distributed ledger mechanisms and incentive mechanisms of blockchains facilitate the reliability of data storage and promote the engagement of data owners [93] in IoV-based operations. The first step in our FC framework is to assess and determine the privacy protection requirements of federated tasks with federated control techniques. We recognize that different tasks may have varying levels of sensitivity when it comes to data privacy. Consequently, the proposed framework tailors its privacy protection mechanisms accordingly. Then, privacy data are encoded and shared with other nodes in the IoV network. This sharing can occur through either decentralized or centralized nodes, depending on the specific needs of the task and the level of trust among participants. With access to information from multiple nodes, FC can be initiated to train intelligent models with prominent approaches such as federated intelligence [94–96] and distributed computing. These techniques enable the collaborative training of models without the need for centralizing sensitive data, thus preserving data privacy and security. To incentivize the sharing of data resources and safeguard the privacy and rights of data owners, our framework includes an incentive mechanism. This mechanism evaluates the value and benefit of information sharing and rewards participants accordingly. By aligning the interests of all stakeholders, we promote a cooperative environment that encourages data sharing for improving autonomous driving systems.

Building upon extensive and diverse driving data resources, we are able to adapt large general models to specific tasks [76] such as vision tasks, social interaction, and risk assessment. Rather than solely focusing on the development of large models explicitly designed for autonomous driving, we advocate for models that encompass a broader knowledge base beyond just the act of driving itself. In other words, the key to establishing the foundation intelligence for autonomous driving lies in strengthening artificial general intelligence (AGI)-like models with domain-specific driving knowledge. To achieve this, we have devised a hierarchical framework that incorporates a two-level adaptation approach, designed to cultivate the foundation intelligence required for autonomous driving. In the first stage of this framework, we facilitate the transfer of general big models into the domain of autonomous driving. This transition is accomplished through the implementation of self-supervised training strategies, which motivate these general models to comprehend the intricacies of autonomous driving inputs. By exposing the models to a variety of data from driving scenarios from the real or virtual world, they learn to interpret and process the relevant information necessary for autonomous driving tasks. Following this transfer, we proceed to fine-tune the models with supervised training techniques. This fine-tuning process is crucial for tailoring the models to specific autonomous driving tasks. Moreover, we design specific model heads tailored to diverse tasks within the autonomous driving domain. These specialized heads are responsible for vision tasks such as object tracking, map generation, trajectory prediction, and motion planning. Besides, heads for social interaction and risk assessment models can also be designed to understand the relationships and situations in the driving environment. By incorporating these specific heads, our models become highly adaptable and capable of handling a wide range of tasks essential for autonomous driving.

## Discussion

### 6S-oriented utility evaluation of parallel driving

**Fig. 6.**
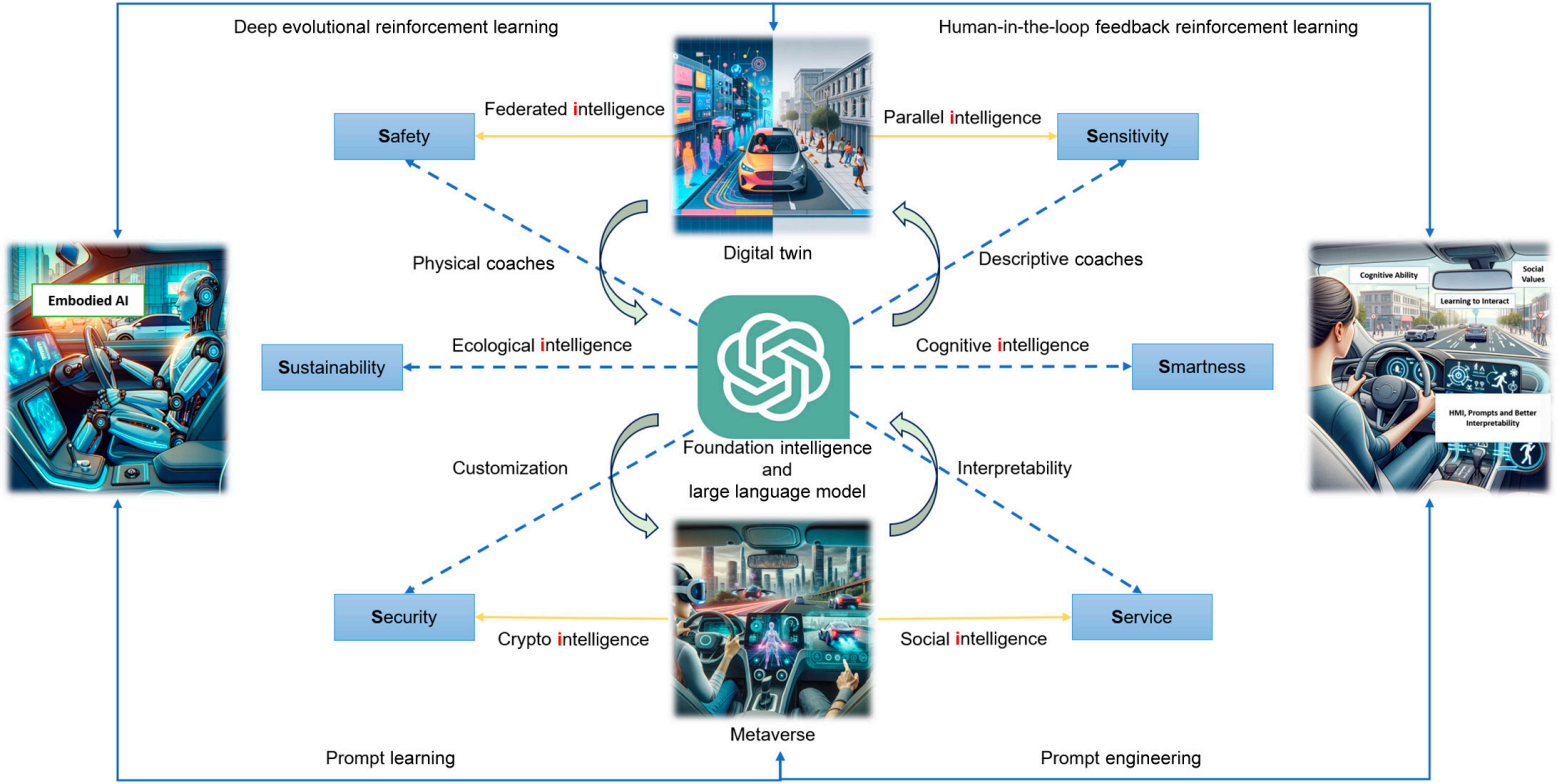
6S-oriented real-time evaluation radar chart of parallel driving.

In this part, we illustrate the 6S objectives (Safety, Security, Sustainability, Sensitivity, Service and Smartness) of Parallel Driving. [97]. A comprehensive framework that integrates these objectives into the core functioning of CPSS is proposed, ensuring a balanced and holistic approach to traffic management and vehicular automation, as can be seen in Fig. 6.

#### Safety

In mixed-traffic situations and hybrid transportation systems, the concept of safety is multifaceted, encompassing both the reduction of traffic accidents and the assurance of physical driving safety. This dual approach to safety is particularly pertinent in environments transitioning to mixed-traffic models with both autonomous and HDVs. The integration of advanced traffic control systems, as explored in the paper, plays a pivotal role in this context. These systems enhance the coordination and management of diverse traffic elements, thereby mitigating collision risks and elevating overall traffic safety.

Quantitatively, safety can be assessed by measuring the accident rate, the number of accidents per million vehicle miles traveled, in both real-world deployment and simulated environments. Aligning these metrics from virtual models to real-world scenarios necessitates a robust correlation methodology, ensuring that simulations accurately reflect on-road conditions. Furthermore, a comprehensive safety analysis should include the recording of near-miss incidents and the effectiveness of safety fallback strategies. These metrics offer critical insights into the preventative capabilities of traffic management systems. Additionally, in considering the implementation of LLMs and other advanced computational methods in traffic systems, it is crucial to evaluate their response times. The speed at which these models process and react to dynamic traffic situations is a key determinant of their efficacy in enhancing road safety. This comprehensive approach to evaluating safety in traffic management not only addresses immediate accident rates but also focuses on the underlying systems and models that inform traffic control decisions, ensuring a thorough and nuanced understanding of safety in mixed-traffic environments.

#### Security

The security aspect focuses on safeguarding digital and operational integrity against potential cyber threats, an area that has gained paramount importance in the age of CAVs. Ensuring the security of traffic systems involves multiple layers of protection against various forms of cyberattacks that could disrupt the seamless operation of these systems. This is particularly crucial in mixed-traffic environments, where the interplay between autonomous vehicles and traditional traffic infrastructure introduces complex security challenges.

A key measure of security within traffic systems is the frequency and severity of security breaches, alongside the responsiveness of the system in detecting and mitigating these threats. For instance, a paramount concern is the susceptibility to distributed denial of service (DDoS) attacks, which could impair real-time communication between vehicles and traffic management systems, potentially leading to significant disruptions. Additionally, the security framework must address the risks of data tampering, where malicious actors could manipulate traffic data to create false scenarios, such as artificial congestion, thereby misleading the traffic management system. Another critical aspect of security in this context is the prevention of unauthorized access, which could lead to significant privacy breaches. This is especially relevant in systems where personal data, such as driver and passenger information, vehicle movements, and behavioral patterns, are communicated between vehicles and traffic infrastructure. Ensuring robust encryption and secure communication channels is essential to maintain confidentiality and integrity.

Moreover, the security of traffic management systems extends beyond immediate cyber threats to include long-term resilience. This involves continuous monitoring, updating, and fortifying of security protocols to stay ahead of evolving cyber threats. In essence, security in traffic management is about not only safeguarding against current threats but also anticipating and preparing for future vulnerabilities, thereby ensuring the ongoing reliability and trustworthiness of the traffic management ecosystem.

#### Sustainability

Sustainability pertains to the environmental and long-term operational impacts of transportation systems. It involves a holistic approach that considers not only the immediate efficiency of traffic flow but also the broader ecological implications. In mixed-traffic environments, particularly those incorporating autonomous vehicles, the potential for enhancing sustainability is significant [98].

The primary indicators of sustainability in traffic systems include the reduction of greenhouse gas emissions and overall energy consumption. Advanced traffic management systems, as discussed in the referenced paper, can contribute to these goals by optimizing traffic flow, thereby reducing idle times and unnecessary stop-and-go conditions at intersections. This optimization directly translates to lower fuel consumption and emissions, particularly in areas with high traffic volume. Simultaneously, another vital component of sustainability in traffic management is the effective handling and reduction of traffic congestion. Congestion is not only a source of increased emissions but also a catalyst for economic inefficiencies, manifesting in prolonged travel durations and escalated operating expenses for transport networks. The adoption of intelligent traffic management solutions, including the deployment of autonomous vehicles and sophisticated traffic signal systems, holds considerable promise in mitigating congestion issues. This reduction in congestion directly improves the operational efficiency of the traffic system, resulting in a decrease in environmental impact and fostering more sustainable urban growth.

Furthermore, sustainability in traffic management also encompasses the resilience and adaptability of the system to future changes. This includes the ability to integrate new technologies, accommodate increasing traffic volumes, and respond to evolving environmental policies and regulations. Sustainable traffic management systems are thus designed with a long-term perspective, ensuring that they remain effective, efficient, and environmentally responsible in the face of future challenges and advancements.

#### Sensitivity

Sensitivity of CAVs in ITS is about ensuring their adaptability to the diverse needs of all users, including drivers, pedestrians, and the community. It goes beyond technical aspects to include social and ethical considerations, aiming for an equitable and inclusive system.

The key to sensitivity is designing traffic strategies that cater to various user behaviors and preferences, especially in mixed environments with autonomous and HDVs. This involves creating systems that are intuitively responsive to each user group, ensuring accessibility and fairness. Moreover, sensitivity encompasses understanding the societal impacts of traffic management, such as job displacement due to automation or changes in public space use. Developing traffic systems with a human-centric approach, involving community engagement and impact assessments, is crucial to mitigate potential negative social effects [99].

In essence, sensitivity in traffic management combines technical proficiency with social awareness, aiming for a system that is not only efficient but also socially attuned and inclusive. This approach helps distribute the benefits of advanced traffic systems equitably, fostering a cohesive and resilient urban community.

#### Service

In mixed traffic, the service aspect of CAVs' emphasizes the user experience and the effectiveness of the system in meeting the needs of all stakeholders. This involves assessing the quality of service provided by the traffic management system from the perspective of drivers, pedestrians, and the broader community.

A critical element of service is the system’s ability to deliver efficient, reliable, and timely traffic management. This includes reducing waiting times at intersections, ensuring smooth traffic flow, and minimizing disruptions. For instance, the integration of intelligent traffic signals and autonomous vehicles, as discussed in the paper, can enhance traffic efficiency, directly improving the user experience. Another important aspect of service is the system’s capability to provide accurate and up-to-date information to users. This might involve real-time traffic updates, alerts about road conditions, or guidance for optimal routes. Effective communication with users, especially in dynamic traffic scenarios, is essential for maintaining a high level of service. Additionally, service quality in traffic management also entails considering the long-term sustainability of the system. This means designing traffic solutions that not only are effective in the short term but also can adapt and evolve with changing user needs and technological advancements.

In summary, service in traffic management focuses on creating a user-centered system that is efficient, informative, and adaptable. It is about ensuring that the traffic management system not only meets the current needs of its users but also is prepared to evolve and improve over time, thereby maintaining a high standard of service in the long run.

#### Smartness

The smartness aspect in mixed traffic is centered on the intelligent and adaptive functioning of the system, particularly in terms of resource utilization, error management, and real-time adaptability to changing conditions. This attribute is crucial in the context of increasingly complex and dynamic traffic environments, especially those integrating autonomous vehicles. A primary indicator of smartness is the system’s efficiency in managing traffic flows. This includes optimizing route allocations, reducing congestion, and effectively handling peak traffic scenarios. The use of advanced algorithms, data analytics, and machine learning techniques plays a significant role in achieving these goals [82]. These technologies enable the traffic system to analyze vast amounts of data, predict traffic patterns, and make informed decisions that enhance overall efficiency. Moreover, error management is another critical aspect of smartness. This involves the system’s capability to detect, diagnose, and rectify issues promptly. In the context of traffic management, this could mean quickly resolving signal malfunctions, addressing traffic jams, or rerouting traffic in response to accidents or road closures. The system’s ability to learn from these incidents and adapt its strategies accordingly is a testament to its smartness.

Lastly, adaptability in real-time conditions is a hallmark of a smart traffic management system. This entails the ability to respond to unexpected changes, such as weather variations, emergency situations, or unusual traffic patterns, and adjust operations seamlessly. The system should be capable of integrating real-time data from various sources, including sensors and user inputs, to make immediate adjustments that maintain smooth traffic flow. In essence, smartness in traffic management encapsulates the system’s ability to function intelligently and adaptively, ensuring efficient, error-free, and responsive operations. It is about leveraging technology to create a traffic system that is not only reactive to current conditions but also proactive in anticipating and adapting to future challenges.

## Conclusion

This article has embarked on an extensive exploration of the integration and embedment of big models, DTs, metaverse, and other emerging technologies in the domain of CAVs within mixed traffic. The principal focus has been on the embodiment of foundation intelligence of parallel driving architectures capable of operating within the complex dynamics of complex environments, significantly influenced by the cyber–physical–social interplay. The innovative approach of embedding big models into CAVs, complemented by advanced edge computing, has showcased the potential for enhancing vehicle intelligence, emphasizing safety, efficiency, reliability, and environmental sustainability. The deployment of MDT and the concept of metamobility have emerged as pivotal elements in creating highly accurate, dynamic representations of the transportation ecosystem. These models, through their real-time synchronization with physical systems, provide a foundation for the predictive intelligence necessary for parallel driving. In parallel, the integration of human-in-the-loop deep evolutional reinforcement learning (DERLHF) with big models has demonstrated an advanced method for refining CAV control strategies, emphasizing the significant role of human expertise and feedback in the continuous improvement of autonomous systems. Furthermore, this study has delved into the critical aspects of safety, security, sustainability, sensitivity, service, and smartness within traffic management—collectively referred to as the “6S” goals. These goals encompass the multifaceted challenges and opportunities presented by the integration of advanced technologies in traffic systems, ranging from ensuring physical safety and digital security to promoting ecological sustainability and social inclusivity.

In conclusion, the investigation into foundation intelligence and FC within the IoV heralds a transformative era in traffic management and the operations of CAVs. The methodologies and frameworks explored in this discourse establish a benchmark for future exploration and innovation in this domain. We aim to cultivate intelligent, safe, and sustainable transportation networks. As we progress, the ongoing advancement and amalgamation of these technologies will be instrumental in defining the future of transportation. This future is characterized by a need for adaptive, intelligent, and “human-centric” strategies in traffic management and vehicular automation. Elevating this vision, we foresee a paradigm where parallel driving becomes the norm in a synergistic parallel space, seamlessly blending human intuition with technological precision to revolutionize our mobility and connectivity.

## Data Availability

There are no data associated with this article.
